# Supplementary material for: A Critical View of the Application of the APEX Software (Aqueous Photochemistry of Environmentally-Occurring Xenobiotics) to Predict Photoreaction Kinetics in Surface Freshwaters
Source: Molecules. 2019 Dec 18;25(1):9. doi: 10.3390/molecules25010009 (PMC7017383; doi:10.3390/molecules25010009)
Supplement: Supplementary file 1 [file molecules-25-00009-s001.zip › 3.2.4_gcc-4.4.0.zip!/3.2.4_gcc-4.4.0/3.2.4_gcc-4.4.0/doc/octave/html/interpreter/Plot-Annotations.html]

Plot Annotations - Untitled


Next: Multiple Plots on One Page,
Previous: Three-Dimensional Plotting,
Up: Plotting Basics


---

#### 15.1.3 Plot Annotations

You can add titles, axis labels, legends, and arbitrary text to an
existing plot. For example,

```
     x = -10:0.1:10;
     plot (x, sin (x));
     title ("sin(x) for x = -10:0.1:10");
     xlabel ("x");
     ylabel ("sin (x)");
     text (pi, 0.7, "arbitrary text");
     legend ("sin (x)");
```

The functions `grid` and `box` may also be used to add grid
and border lines to the plot. By default, the grid is off and the
border lines are on.

— Function File: **title** (title)  
— Function File: **title** (title, p1, v1, ...)  
> Create a title object and return a handle to it.

— Function File: **legend** (st1, st2, ...)  
— Function File: **legend** (st1, st2, ..., "location", pos)  
— Function File: **legend** (matstr)  
— Function File: **legend** (matstr, "location", pos)  
— Function File: **legend** (cell)  
— Function File: **legend** (cell, "location", pos)  
— Function File: **legend** ('func')  
> Display a legend for the current axes using the specified strings
> as labels. Legend entries may be specified as individual character
> string arguments, a character array, or a cell array of character
> strings. Legend works on line graphs, bar graphs, etc. A plot must
> exist before legend is called.
>
> The optional parameter pos specifies the location of the legend
> as follows:
>
> |  |  |  |
> | --- | --- | --- |
> |  | north | center top |
> |  | south | center bottom |
> |  | east | right center |
> |  | west | left center |
> |  | northeast | right top (default) |
> |  | northwest | left top |
> |  | southeast | right bottom |
> |  | southwest | left bottom |
> |  |  |  |
> |  | outside | can be appended to any location string |
>
> Some specific functions are directly available using func:
>
> "show": Show legends from the plot "hide" "off": Hide legends from the plot "boxon": Draw a box around legends "boxoff": Withdraw the box around legends "left": Text is to the left of the keys "right": Text is to the right of the keys

— Function File: h = **text** (x, y, label)  
— Function File: h = **text** (x, y, z, label)  
— Function File: h = **text** (x, y, label, p1, v1, ...)  
— Function File: h = **text** (x, y, z, label, p1, v1, ...)  
> Create a text object with text label at position x,
> y, z on the current axes. Property-value pairs following
> label may be used to specify the appearance of the text.

See Text Properties for the properties that you can set.

— Function File: **xlabel** (string)  
— Function File: **ylabel** (string)  
— Function File: **zlabel** (string)  
— Function File: **xlabel** (h, string)  
> Specify x, y, and z axis labels for the current figure. If h is
> specified then label the axis defined by h.
>
>
>
> **See also:** plot, semilogx, semilogy, loglog, polar, mesh, contour, bar, stairs, title.

— Function File: **clabel** (c, h)  
— Function File: **clabel** (c, h, v)  
— Function File: **clabel** (c, h, "manual")  
— Function File: **clabel** (c)  
— Function File: **clabel** (c, h)  
— Function File: **clabel** (..., prop, val, ...)  
— Function File: h = **clabel** (...)  
> Adds labels to the contours of a contour plot. The contour plot is specified
> by the contour matrix c and optionally the contourgroup object h
> that are returned by `contour`, `contourf` and `contour3`.
> The contour labels are rotated and placed in the contour itself.
>
> By default, all contours are labelled. However, the contours to label can be
> specified by the vector v. If the "manual" argument is given then
> the contours to label can be selected with the mouse.
>
> Additional property/value pairs that are valid properties of text objects
> can be given and are passed to the underlying text objects. Additionally,
> the property "LabelSpacing" is available allowing the spacing between labels
> on a contour (in points) to be specified. The default is 144 points, or 2
> inches.
>
> The returned value h is the set of text object that represent the
> contour labels. The "userdata" property of the text objects contains the
> numerical value of the contour label.
>
> An example of the use of `clabel` is
>
> ```
>           [c, h] = contour (peaks(), -4 : 6);
>           clabel (c, h, -4 : 2 : 6, 'fontsize', 12);
> ```
>
>
>
>
> **See also:** contour, contourf, contour3, meshc, surfc, text.

— Function File: **box** (arg)  
— Function File: **box** (h, ...)  
> Control the display of a border around the plot.
> The argument may be either `"on"` or `"off"`. If it is
> omitted, the current box state is toggled.
>
>
>
> **See also:** grid.

— Function File: **grid** (arg)  
— Function File: **grid** ("minor", arg2)  
— Function File: **grid** (hax, ...)  
> Force the display of a grid on the plot.
> The argument may be either `"on"`, or `"off"`.
> If it is omitted, the current grid state is toggled.
>
> If arg is `"minor"` then the minor grid is toggled. When
> using a minor grid a second argument arg2 is allowed, which can
> be either `"on"` or `"off"` to explicitly set the state of
> the minor grid.
>
> If the first argument is an axis handle, hax, operate on the
> specified axis object.
>
>
>
> **See also:** plot.

— Function File: **colorbar** (s)  
— Function File: **colorbar** ("peer", h, ...)  
> Adds a colorbar to the current axes. Valid values for s are
>
> "EastOutside": Place the colorbar outside the plot to the right. This is the default. "East": Place the colorbar inside the plot to the right. "WestOutside": Place the colorbar outside the plot to the left. "West": Place the colorbar inside the plot to the left. "NorthOutside": Place the colorbar above the plot. "North": Place the colorbar at the top of the plot. "SouthOutside": Place the colorbar under the plot. "South": Place the colorbar at the bottom of the plot. "Off", "None": Remove any existing colorbar from the plot.
>
> If the argument "peer" is given, then the following argument is treated
> as the axes handle on which to add the colorbar.
